# Root Rot Management in Common Bean (*Phaseolus vulgaris* L.) Through Integrated Biocontrol Strategies using Metabolites from *Trichoderma harzianum*, *Serratia marcescens*, and Vermicompost Tea

**DOI:** 10.1007/s00248-024-02400-4

**Published:** 2024-07-15

**Authors:** Karima G. Helmy, Samah H. Abu-Hussien

**Affiliations:** 1 Plant Pathology Department, Faculty of Agriculture, Ain Shams University, Cairo, 11241 Egypt; 2 Agricultural Microbiology Department, Faculty of Agriculture, Ain Shams University, Cairo, 11241 Egypt

**Keywords:** Root rot, Common bean, Biological control, Vermicompost tea, Integrated disease management

## Abstract

Common bean (*Phaseolus vulgaris* L.) is an essential food staple and source of income for small-holder farmers across Africa. However, yields are greatly threatened by fungal diseases like root rot induced by *Rhizoctonia solani*. This study aimed to evaluate an integrated approach utilizing vermicompost tea (VCT) and antagonistic microbes for effective and sustainable management of *R. solani* root rot in common beans. Fourteen fungal strains were first isolated from infected common bean plants collected across three Egyptian governorates, with *R. solani* being the most virulent isolate with 50% dominance. Subsequently, the antagonistic potential of vermicompost tea (VCT), *Serratia* sp., and *Trichoderma* sp. was assessed against this destructive pathogen. Combinations of 10% VCT and the biocontrol agent isolates displayed potent inhibition of *R. solani* growth in vitro, prompting in planta testing. Under greenhouse conditions, integrated applications of 5 or 10% VCT with *Serratia marcescens*, *Trichoderma harzianum*, or effective microorganisms (EM1) afforded up to 95% protection against pre- and post-emergence damping-off induced by *R. solani* in common bean cv. Giza 6. Similarly, under field conditions, combining VCT with EM1 (VCT + EM1) or *Trichoderma harzianum* (VCT + *Trichoderma harzianum*) substantially suppressed disease severity by 65.6% and 64.34%, respectively, relative to untreated plants. These treatments also elicited defense enzyme activity and distinctly improved growth parameters including 136.68% and 132.49% increases in pod weight per plant over control plants. GC–MS profiling of *Trichoderma harzianum*, *Serratia marcescens*, and vermicompost tea (VCT) extracts revealed unique compounds dominated by cyclic pregnane, fatty acid methyl esters, linoleic acid derivatives, and free fatty acids like oleic, palmitic, and stearic acids with confirmed biocontrol and plant growth-promoting activities. The results verify VCT-mediated delivery of synergistic microbial consortia as a sustainable platform for integrated management of debilitating soil-borne diseases, enhancing productivity and incomes for smallholder bean farmers through regeneration of soil health. Further large-scale validation can pave the adoption of this climate-resilient approach for securing food and nutrition security.

## Introduction

Common bean (*Phaseolus vulgaris* L.) is a vital food security crop and source of income for smallholder farming communities across Africa [1]. As a rich source of proteins, vitamins, minerals, and phytonutrients, it provides affordable nutrition to impoverished households [2]. Moreover, the nitrogen-fixing property allows it to improve soil fertility in intercropping systems alongside cereals like maize and sorghum [3]. However, bean productivity across sub-Saharan Africa continues to remain under two tons per hectare—nearly half the estimated potential—negatively impacting the incomes and livelihoods of farmers [4].

Among the various production challenges, fungal diseases pose persistent and devastating threats. Root rot complex resulting from infection by soil-borne pathogens like *Fusarium *sp., *Rhizoctonia *sp., *Pythium *sp., and *Macrophomina *sp. inflicts huge losses, with yield reductions reaching 100% under conducive conditions. Damping-off, stunted growth, wilts, rots, and vascular discoloration are common symptoms that severely diminish plant stand, biomass, quality, and yields [5]. Changing climatic patterns like rising temperatures, erratic rainfalls, and droughts are further exacerbating disease severity and distribution ranges.

In the absence of genetic resistance, farmers rely extensively on chemical fungicides for managing root rots. However, their rising costs coupled with associated health and environmental hazards due to uncontrolled use make this approach practically and ecologically unsustainable [6]. This underscores the urgency for affordable, safe, and effective alternative solutions for resource-poor smallholders. Biological control utilizing beneficial microorganisms offers an eco-friendly, climate-resilient strategy by reinforcing the native soil microbiome to suppress pathogens through competitive, antagonistic, and plant-mediated interactions [7].

Several bacteria, fungi, and actinomycetes inhabiting the rhizosphere and plant interiors have shown promising biocontrol potential against fungal bean pathogens. Fluorescent pseudomonads producing antifungal metabolites, siderophores, and hydrolytic enzymes can protect plants through direct inhibition of pathogens including *Fusarium* sp., *Rhizoctonia* sp., *Pythium* sp., and *Sclerotium* sp. [8, 9]. Plant growth-promoting and biocontrol traits are also elicited by *Bacillus* sp. similarly enriched in bean rhizospheres, while non-pathogenic binucleate *Rhizoctonia* sp. strains demonstrate biocontrol activity through mycoparasitism and induced resistance pathways [10]. Likewise, *Trichoderma* sp. fungi prolifically generate lytic enzymes and antibiotics that dually impede phytopathogens through mycoparasitism and vitalize plant growth and defenses [11]. However, inconsistencies in field efficacy, viability, and environmental fitness of introduced bioagents pose adoption challenges.

Therefore, utilizing organic amendments like vermicomposts to sustain native as well as augmented biocontrol consortia offers a pragmatic solution [12]. Vermicomposts not only provide a buffered microenvironment but also supply microbial substrates through the dynamic mineralization of nutrients bound up in the intricate matrices of partially decomposed organic residues and waste products [13]. Water-soluble extracts referred to as vermicompost teas (VCT) allow practical field application for mobilizing beneficial rhizosphere communities, directly competing with and antagonizing pathogens [14]. The teas also elicit growth and systemic defenses in treated plants against diseases through unique blends of nutrient availability, microbial metabolites, and signaling compounds [15, 16]. This study aimed to develop an integrated biological management strategy for controlling *Rhizoctonia* root rot in common beans using synergistic combinations of VCT and antagonistic microbes like *Trichoderma* sp., *Serratia* sp., and effective microorganisms (EM1). The treatments were tested under both greenhouse and field conditions to validate their efficacy for farmer-level adoption and strengthening resilience in smallholder bean production systems.

## Methods

### Plant Material

Common bean (*Phaseolus vulgaris* L.) seeds of the commercial cultivar Giza 6 susceptible to root rot were obtained from the Agricultural Research Center, Giza, Egypt, and used across experiments.

### Isolation of Root Rot Fungi

**Fig. 1:**
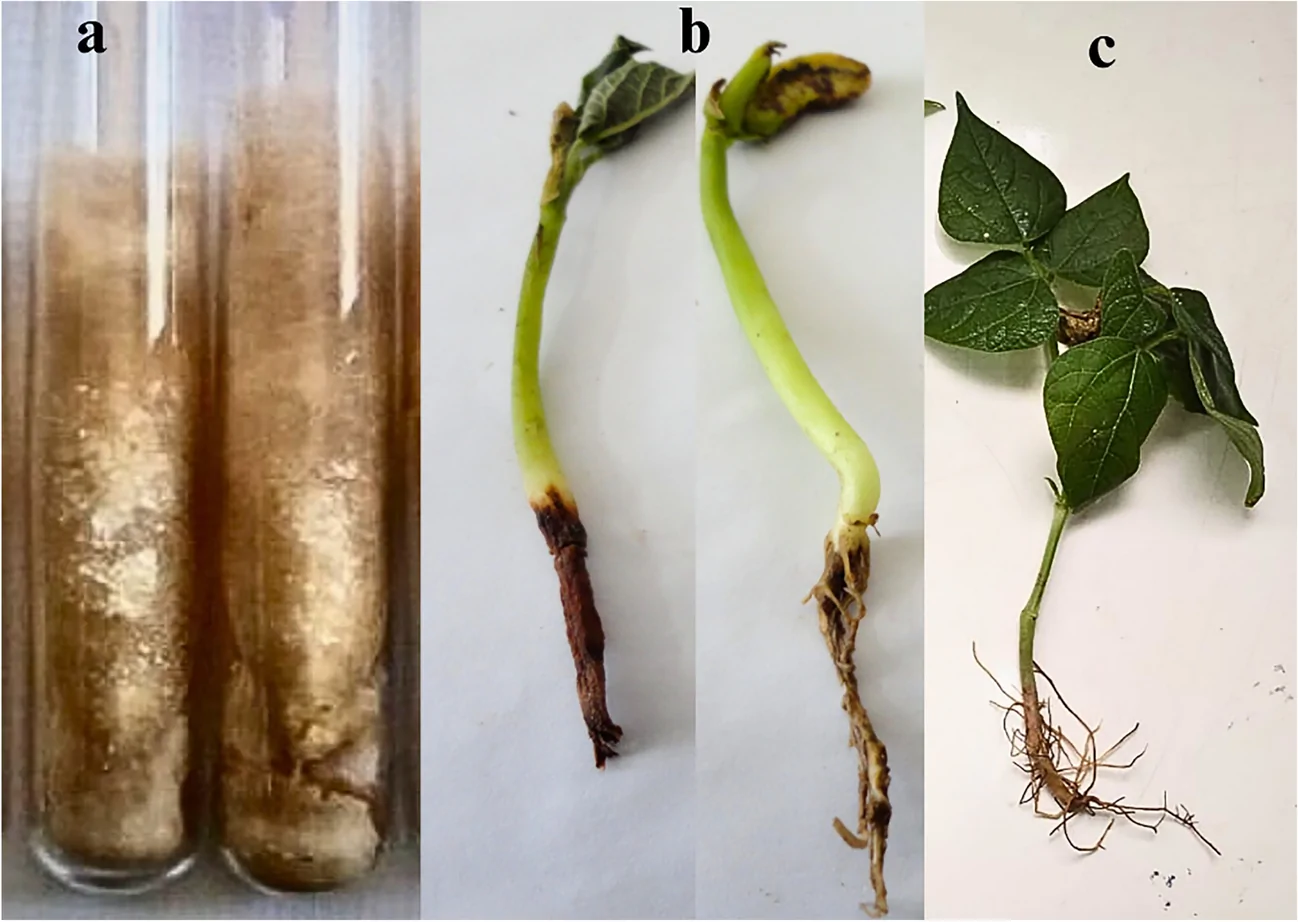
Typical natural symptoms of common bean root rot on common bean plant behind healthy one. **a**: Pure culture of *R. solani* isolate grew on PDA medium. **b**: The black microsclerotia are visible on the root and the lower part of the stem of the diseased plant. **c**: Healthy common bean plant (control)

Infected common bean root samples displaying symptoms of rotting, discoloration, and reduced growth **(**Fig. 1**)** were collected during the flowering stage from major bean cultivation areas in Giza, Sharqia, and Qalyubia governorates. The infected roots were surface sterilized with 2% sodium hypochlorite solution for 2 min [17], rinsed in sterile water, air dried, and cut into segments then plated on potato dextrose agar medium (PDA, pH 5.6 ± 0.2 containing infusion from 200 g potatoes, 20 g dextrose, 15 g agar, and distilled water up to 1 L) [18]. Plates were incubated at 25 °C for 5–7 days, and emerging fungal colonies were subsequently purified by hyphal tip subculturing on fresh PDA every 15 days and maintained at 4 °C for identification and downstream experiments [19]. After purification, the frequency of the *R. solani* fungus was calculated with the following Eq. 1:

1 $$Frequency\;of\;Pathogens\;\%=\frac{Number\;of\;total\;isolated\;pathogens-infected\;samples}{Total\;number\;of\;samples\;tested}\times100$$

### Microorganisms Used

Bacterial isolate *Serratia* S1 was isolated from the rhizosphere of healthy common bean plants growing in fields infested with root rot disease and was streaked on tryptic soy agar (TSA, pH 7.0 ± 0.2 with casein peptone, soybean peptone, sodium chloride, dipotassium phosphate, and dextrose) for purification [20]. The red-pigmented colonies were picked up, repeatedly subcultured on fresh TSA slants every week, and stored at 4 °C. Standard inoculum was prepared by inoculating 50 mL TSB (tryptic soy broth without agar) and incubating overnight at 30 °C with shaking at 150 rpm. The culture was centrifuged at 10,000 g for 10 min, and the pellet was resuspended in sterile distilled water and adjusted to 10^8^ CFU/mL using 0.5 McFarland standard for further antagonist testing [20].

*Trichoderma* sp. was isolated from a soil sample collected from the rhizosphere of common bean plants. The fungus was picked up and purified by subculturing on a potato dextrose agar (PDA) medium. For standard inoculum preparation, *Trichoderma* sp. was grown on PDA at 25 ± 1 °C for 10 days for conidia production. The fungal spores were then scraped and resuspended in sterile water to make a conidial suspension, filtered through cheesecloth, and quantified by hemocytometer to obtain a final stock suspension of 3 × 10^6^ conidia/mL in water [11].

#### Effective Microorganisms (EM1)

The commercial product EM1 containing lactic acid bacteria, yeasts, and phototrophic bacteria was acquired from the Ministry of Agriculture, El-Dokki, Egypt, under license from the Eastern Mediterranean Regional Office, Japan.

### Preparation of Vermiculite Compost Tea (VCT)

Vermiculite compost tea (VCT) was obtained from the Central Laboratory for Agricultural Climate, El Dokki, Giza, Egypt. Briefly, VCT was prepared by mixing high quality vermicompost with non-chlorinated water in a ratio of 1:10 (w/v). The mixture was incubated at 25 °C for 7 days with intermittent stirring on day 4 using an aquarium pump. The VCT was filtered through a sieve before use to remove large vermicompost particles. After that, the obtained VCT was diluted with water at 5 and 10% ((w/v) just before application to plants). VCT was stored in a cool, dark place during brewing and was used within a few days for optimal results [16].

#### The Fungicide Rizolex-T

Fifty percent containing tolclofos-methyl and thiram active ingredients was obtained from the Egyptian Ministry of Agriculture.

### Pathogenicity Test

The pathogenicity of the isolated fungal species (*R. solani*, *F. solani*, *M. phaseolina*, and *Pythium* sp.) was tested on the common bean cv. Giza 6 under greenhouse conditions. Inoculum of each fungal isolate was prepared by cultivating it on a sorghum-sand medium. Pots (30 cm) were filled with a mixture of autoclaved sand and soil, infested individually with 3% (w/w) of the inoculum of each fungal isolate, and left for 7 days after thorough irrigation. Five common bean seeds were then sown in each pot per isolate. Disease incidence in terms of pre-emergence damping off (%), post-emergence damping off (%), and plant survival rate was recorded. The most aggressive isolate of *R. solani* obtained from the pathogenicity test was selected for further in vitro and in vivo studies [21, 22].

### Identification of Bacterial and Fungal Isolates

#### Phenotypic Identification

All obtained isolates were identified according to their cultural and cell morphological characteristics. Gram staining was applied to bacterial isolates [20]. Slide cultures were applied to fungal isolates [23].

### Molecular Identification by 16s rDNA Gene Analysis

#### Bacterial Genotypic Identification

Bacterial identification was performed by extracting bacterial DNA and using polymerase chain reaction (PCR) to amplify the 16S ribosomal RNA gene region. Universal primers (forward primer F1, 5′ AGAGTTTGATCCTGGCTCAG 3′; reverse primer R1, 5′ ACGGCTACCTTGTTACGACTT 3′) targeted conserved sites flanking this region to generate amplicons for sequencing. After PCR purification, samples were sent for Sanger sequencing of the 16S rRNA gene. The resulting reads were computationally trimmed, assembled, and aligned before using BLAST similarity searches to identify taxa. Phylogenetic trees were constructed with neighbor-joining cluster analysis to visualize evolutionary relationships [20].

#### Fungal Genotypic Identification

For fungal identification, cultures were sent for 18S ribosomal RNA gene sequencing. Fungal DNA extraction preceded PCR targeting the internal transcribed spacer (ITS) region. Universal ITS1 and ITS4 primers (Forward primer (ITS1), 5′-TCCGTAGGTGAACCTGCGG-3′; reverse primer (ITS4), 5′-TCCTCCGCTTATTGATATGC-3′) amplified this sequence with incorporated ddNTPs. Like the bacterial amplicons, purified fungal PCR products were Sanger sequenced, and the reads were processed through trimming, BLAST annotation, and phylogenetic tree building to deduce taxa and evolutionary histories [24].

#### Antagonistic Activity of the Isolated Bacterial Bioagents

The purified bacterial strains underwent evaluation for their antagonistic effects against *R. solani* using well diffusion method [24]. In this procedure, the *Rhizoctonia* mycelium was streaked onto a Petri dish, wells were made using a 7-mm diameter corkporer. Different concentrations (25%, 50%, 100) of the *Serratia*, *Trichoderma* metabolites, and VCT were prepared, then each well was filled with 100 µL of the metabolite concentrations against distilled water as negative control. The plates were subsequently incubated for 5–7 days at 25 ± 2 °C. Following the incubation period, the inhibition zone diameter (cm) was evaluated. Each treatment was replicated three times throughout the experiment.

#### Bioagent Trials Under Greenhouse Conditions

Common bean cv. Giza 6 seeds were soaked in the tested suspension (VCT, EM1, *Trichoderma harzianum*) inoculum individually for 2 h before planting, and then 10 mL of the suspension was added to each pot after 4, 8, 14, and 18 days of planting. The experiment was laid out in a completely randomized design with 14 treatments and 5 replications. Seeds soaked in respective formulations were sown (5 seeds/pot), and treatment applications were repeated weekly till harvest. Emergence rate, survival percentage, and disease severity on a 1–9 scale were recorded after 25 days and analyzed through ANOVA (*P* ≤ 0.05) followed by Duncan’s test for means separation [25].

For VCT application, VCT was incorporated into the soil by adding it to the irrigation water at 5 and 10% (v/w) per pot right after planting and subsequently at 4, 8, 14, and 18 days. For EM1 application, common bean seeds were soaked in an EM1 suspension for 15 min before sowing. Thereafter, EM1 was applied by drenching the pots with 13 mL suspension per pot at 4, 8, 14, and 18 days after planting. The fungicide Rizolex-T was used as a positive control; it was applied by soaking common bean seeds in 1.5 g per kg seed of Rizolex-T for 5 min before sowing. The fungicide soil drench was applied three times at weekly intervals throughout the growing period [25]. Each treatment was replicated three times throughout the experiment.

#### Bioagents Trials Under Field Conditions

For greenhouse trials, common bean seeds were soaked in the test suspensions (VCT, EM1, *Trichoderma harzianum* inoculum) individually for 2 h before planting. After planting, more suspension (10 mL) was added per pot at 4, 8, 14, and 18 days to ensure sufficient inoculation of the seed/seedlings. The experiment was laid out in a completely randomized design. Each treatment plot (3 × 3 m2) comprised 3 rows planted at 60 × 20 cm spacing (2 seeds/hill). Agronomic data including yield attributes were documented at harvest, and disease protection efficacy was determined. For VCT application, VCT was added through irrigation water at 12 L per feddan immediately after planting. This treatment was repeated three more times at biweekly intervals. For EMI application, common bean cv. Giza 6 seeds were soaked in the EM1 suspension for 15 min followed by soil drenching with EM1 at 4 L per feddan along with irrigation water. This treatment was repeated three times at biweekly intervals. The fungicide Rizolex-T was used as a positive control; it was applied by soaking common bean seeds in 1.5 g per kg seed of Rizolex-T for 5 min before sowing. The fungicide soil drench was applied three times at weekly intervals throughout the growing period [25]. Each treatment was replicated three times throughout the experiment.

### Experimental Design Under Greenhouse Conditions

Pot experiments were set up in the greenhouse of the Faculty of Agriculture, Ain Shams University. Pots (30 cm) were filled with a mixture of autoclaved sand and soil. The soil was infested with *R. solani* inoculum at a 3% rate, 1 week before sowing common bean seeds. The experiment comprised 13 treatments including different combinations of vermicompost tea (VCT), effective microorganisms (EM1), *Trichoderma harzianum*, and *Serratia marcescens* along with appropriate controls—*R. solani* infested soil without amendments, autoclaved uninfested soil, and fungicide (Rizolex-T 50%) treatment. The 13 treatment conditions were as follows:

- T1, 5% VCT (v/w); T2, EM1; T3, *Trichoderma harzianum*; T4, *Serratia marcescens*; T5, 5% VCT + EM1; T6, 5% VCT + *Trichoderma harzianum*; T7, 5% VCT + *Serratia marcescens*; T8, 10% VCT; T9, *Trichoderma harzianum*; T10, *Serratia marcescens*; T11, 10% VCT + EM1; T12, 10% VCT + *Trichoderma harzianum*; and T13, 10% VCT + *Serratia marcescens* [25].

### Experimental Design Under Field Conditions

Field trials were conducted for two successive growing seasons in 2018 and 2019 at a site in Sharkia Governorate, Egypt, where common bean root rot disease was prevalent naturally. The experiment comprised 7 treatments including different combinations of vermicompost tea (VCT), effective microorganisms (EM1), *Trichoderma harzianum*, and *Serratia marcescens* along with fungicide (Rizolex-T 50%) control and untreated control without any amendments. The 7 treatments were as follows: T1, VCT; T2, EM1; T3, *Trichoderma harzianum*; T4, *Serratia marcescens*; T5, VCT + EM1; T6, VCT + *Trichoderma harzianum*; and T7, VCT + *Serratia marcescens*. The field was arranged in a randomized complete block design with three replications per treatment. Each plot measured 3.0 × 3.0 m.^2^ with 3 rows and a sowing density of 2 seeds per hill at 15–20 cm spacing. Growth parameters like plant height, root and shoot length, root and shoot fresh and dry weights, and yield attributes such as pods per plant were determined at harvest for analysis [25].

### Disease Assessment

Assessment of disease incidence as percent pre-emergence damping-off, post-emergence damping-off, and surviving plants. Disease severity in terms of root and hypocotyl rot was scored on a 1–9 scale [25, 26], where (1), no symptoms; (3), mild color change with no necrotic lesions or with about 10% lesions on hypocotyl tissues and roots; (5), the presence of 25% lesions on the hypocotyl and root tissues, with some deterioration in the root system; (7), about 50% of root and hypocotyl tissues are covered with lesions with softening, rotting, and reduction in root structure; and (9), about 75% or more of the root and hypocotyl tissues were affected by advanced stages of rot, with significant destruction of the root structure. The percentage of treatment efficiency in the reduction of root rot severity was calculated using the following Eq. 2 [27]:

2 $$Efficiency\, (\%) = \dfrac{Control-Treatment }{Control }x100$$

### Enzyme Extraction

Fresh common bean leaf samples were collected from each treatment and ground to a homogenate using a prechilled mortar and pestle in an ice bath. Extraction was performed using cold 0.1 M potassium phosphate buffer (pH 7.4) containing 3 µL 2-mercaptoethanol at 2 mL buffer per 0.5 g tissue ratio. The homogenate was centrifuged at 10,000 rpm at 4 °C for 20 min, and the resulting supernatant was used as crude enzyme extract to assay peroxidase (POX) and polyphenol oxidase (PPO) activities [28].

#### Peroxidase (POX) Enzyme

Peroxidase (POX) activity was determined by the guaiacol oxidation method. Briefly, the assay mixture contained 10 µL 1% (v/v) guaiacol, 10 µL 0.3% H2O2, and 80 µL 50 mM phosphate buffer (pH 6.6) making up to 100 µL total volume. The reaction was started by adding 100 µL of crude enzyme extract to 2.9 mL of assay mix, and the change in absorbance at 470 nm was recorded every 30 s for 3 min using a UV–Vis spectrophotometer (T60, PG Instruments). The rate of absorbance change per minute was used to calculate enzyme activity. One unit of POX activity was defined as the change in absorbance (ΔOD) of 0.01 per minute. Results were expressed as units per gram fresh weight of leaf tissue. The key reagents, proportions, and readings have been retained while simplifying the terminology and phrasing [29].

#### Polyphenol Oxidase (PPO) Enzyme

Polyphenol oxidase (PPO) activity was determined by the catechol oxidation method. The reaction mixture contained 100 µL of crude enzyme extract, 600 µL of catechol substrate, and 2.3 mL of 0.1 M phosphate buffer (pH 6.5). The increase in absorbance at 420 nm was recorded at zero time and after 1 min using a UV–Vis spectrophotometer (T60, PG Instruments). One unit of PPO activity was defined as the amount causing an increase of 0.001 absorbance units per minute at 420 nm under the assay conditions. The final enzyme activity was expressed as units per gram of fresh weight of leaf tissue [29].

#### Gas Chromatography (GC/MS) Analysis

Extracted metabolites of *T. haharzianum*, *S. marcescens*, and VCT were dried over anhydrous Na_2_SO_4_ using a rotary evaporator and then dissolved by methanol. A capillary column TG-5MS (30 m × 0.25 mm × 0.25 m film thickness) and a Trace GC-TSQ mass spectrometer (Thermo Scientific, Austin, TX, USA) were used. Column temperature was maintained at 50 °C and raised by 5 °C/min until reaching 250 °C then maintained for 2 min expanded by 30 °C/min to an ultimate temperature of 300 °C and held for 2 min. Helium was employed as the carrier gas, with a constant flow rate of 1 mL/min, and temperatures of the injector and MS transfer line were maintained at 270 and 260 °C, respectively. Autosampler AS1300 paired with GC in split mode automatically injected diluted samples of 1 µL with a solvent delay of 4 min. EI mass spectra were collected at 70 eV ionization voltages over the range of 50–650 m/z in full scan mode. The ion source temperature was set at 200 °C. The components were identified by comparison of their mass spectra with those of WILEY 09 and NIST 14 mass spectral database [30].

### Statistical Analysis

The experimental data was statistically analyzed by analysis of variance (ANOVA) using the general linear model (GLM) procedure of SAS software version 9.2 (SAS Institute Inc. 2012). Means were separated by Duncan’s multiple range test at *P* ≤ 0.05 level (Duncan 1955) to determine significant differences between treatments [31].

## Results

### Isolation of Common Bean Root Rot Fungi

**Fig. 2:**
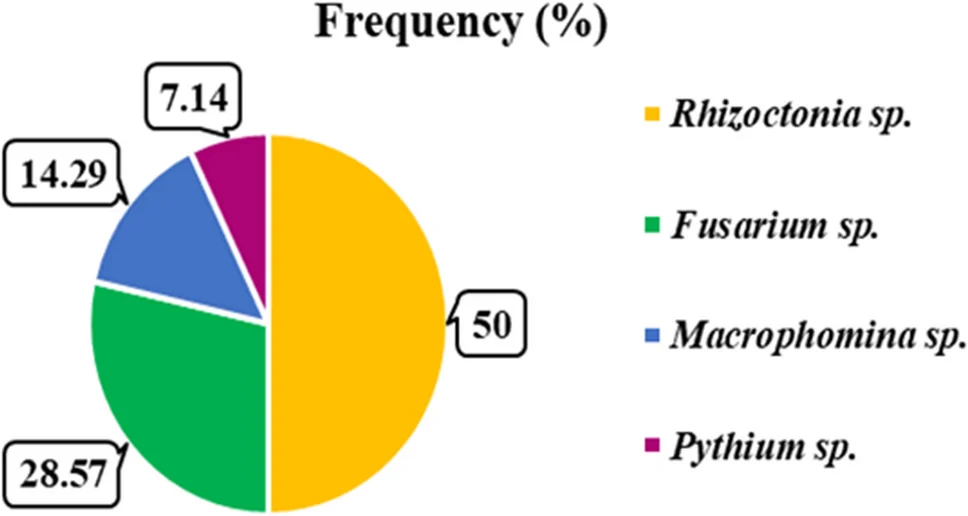
The abundance of isolated fungi (%) from common bean plants infected with damping off and root rot

Fourteen fungal strains were isolated from infected common bean cv. Giza 6 seedlings and roots exhibiting damping-off and root rot symptoms collected from the Giza, Sharqia, and Qalyubia governorates of Egypt** (**Fig. 2). The isolation frequency of the obtained fungi was determined as a percentage of total isolates. *Rhizoctonia sp.* was the predominant isolate with the highest frequency of 50%, followed by *Fusarium* sp. at 28.57%. *Macrophomina* sp. and *Pythium* sp. were isolated at lower frequencies of 14.29% and 7.14%, respectively.

### Pathogenicity Test

**Fig. 3:**
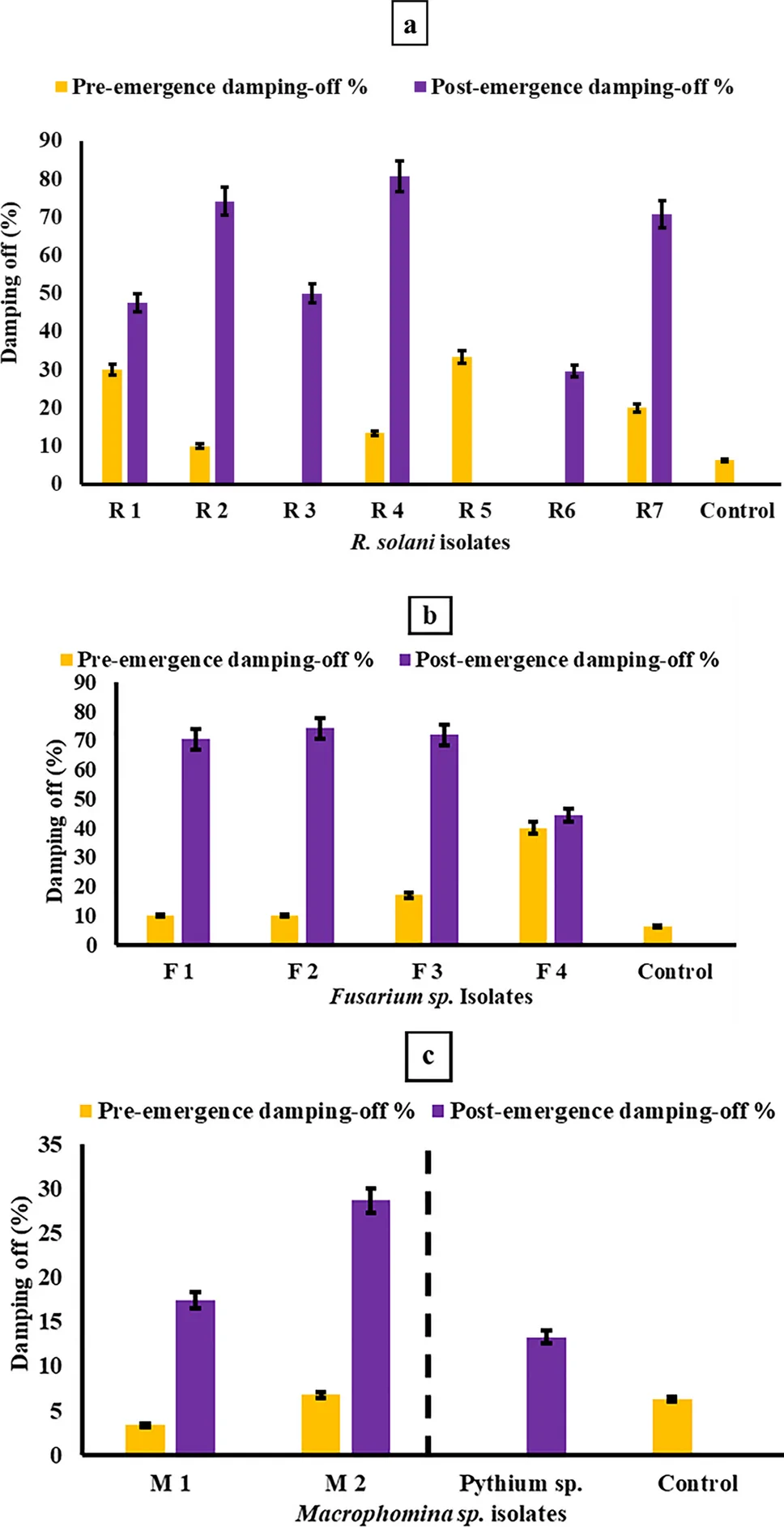
Pathogenicity of fungal isolates from infected common bean cv. Giza 6 plants in terms of pre- and post-emergence damping-off percentage. **a**: *Rhizoctonia* sp. isolates, **b**:*Fusarium* sp. isolates, **c**: *Macrophomina* sp. isolates

Pathogenicity assays were performed by artificially inoculating different fungal isolates obtained from infected common bean plants on healthy cv. Giza 6 seedlings under controlled conditions. Disease incidence as a percentage of pre- and post-emergence damping-off was recorded (Fig. 3) to identify the most aggressive isolates. *F. solani* isolate F4 and *R. solani* isolate R1 caused maximum pre-emergence mortality with 40% and 30% seedling death, respectively, while *R. solani* R3, *R. solani* R6, and *Pythium* sp. isolates resulted in no pre-emergence damping-off. For post-emergence infection, *R. solani* R4 isolate showed the highest virulence with 80.8% damping-off, followed by 74.13% for *R. solani* R2 and *F. solani* F2 isolates. Based on the overall disease severity, *R. solani* R4 isolate was selected as the most aggressive isolate for subsequent antagonistic studies.

### Antagonistic Activity of the VCT and Isolated Bacterial Bioagents

**Fig. 4:**
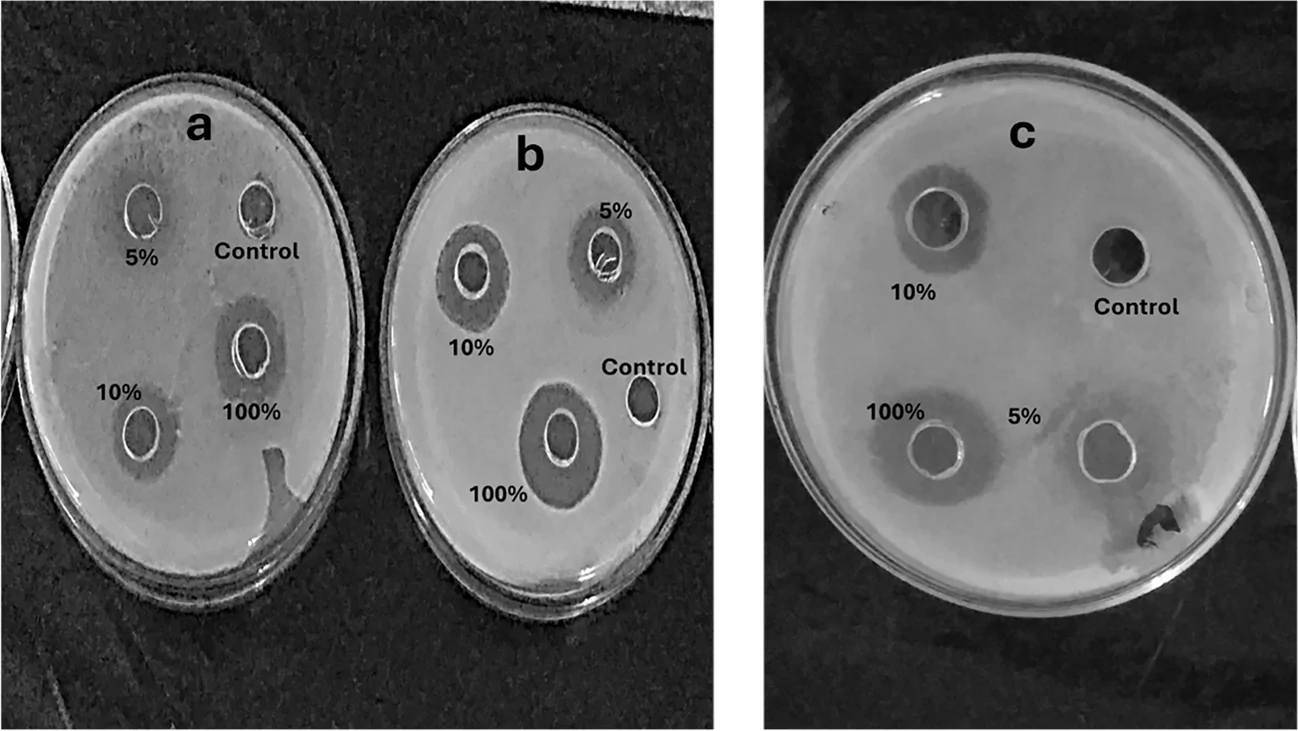
In vitro antagonistic activity of **a**: *Serratia S1 isolate*, **b**: *Trichoderma T15 isolate*, and **c**:VCT extract against *R. solani* after incubation at 25 ± 2℃ for 5–7 days expressed as IZD (cm)

The antagonistic activity of VCT and the isolated bacterial bioagents (Fig. 4) were assessed through in vitro tests using *Trichoderma* sp, *Serratia* sp. metabolites, and VCT which were evaluated for their effectiveness against *Rhizoctonia* R4 isolate. Among these strains, *Serratia* S1 and *Trichoderma* T15 exhibited notable antagonistic effects against the tested phytopathogen (*R. solani*) as illustrated in Fig. 4. In comparison to metabolites of *Serratia* S1 and *Trichoderma* T15 isolates, VCT extract proved a strong antagonistic potential against *Rhizoctonia* R4 isolate’s growth reaching inhibition zone diameters (IZD) of 3.5 and 2.45 cm at 10 and 5%, respectively..

### Identification of Bacterial and Fungal Isolates

#### Phenotypic Identification

**Fig. 5:**
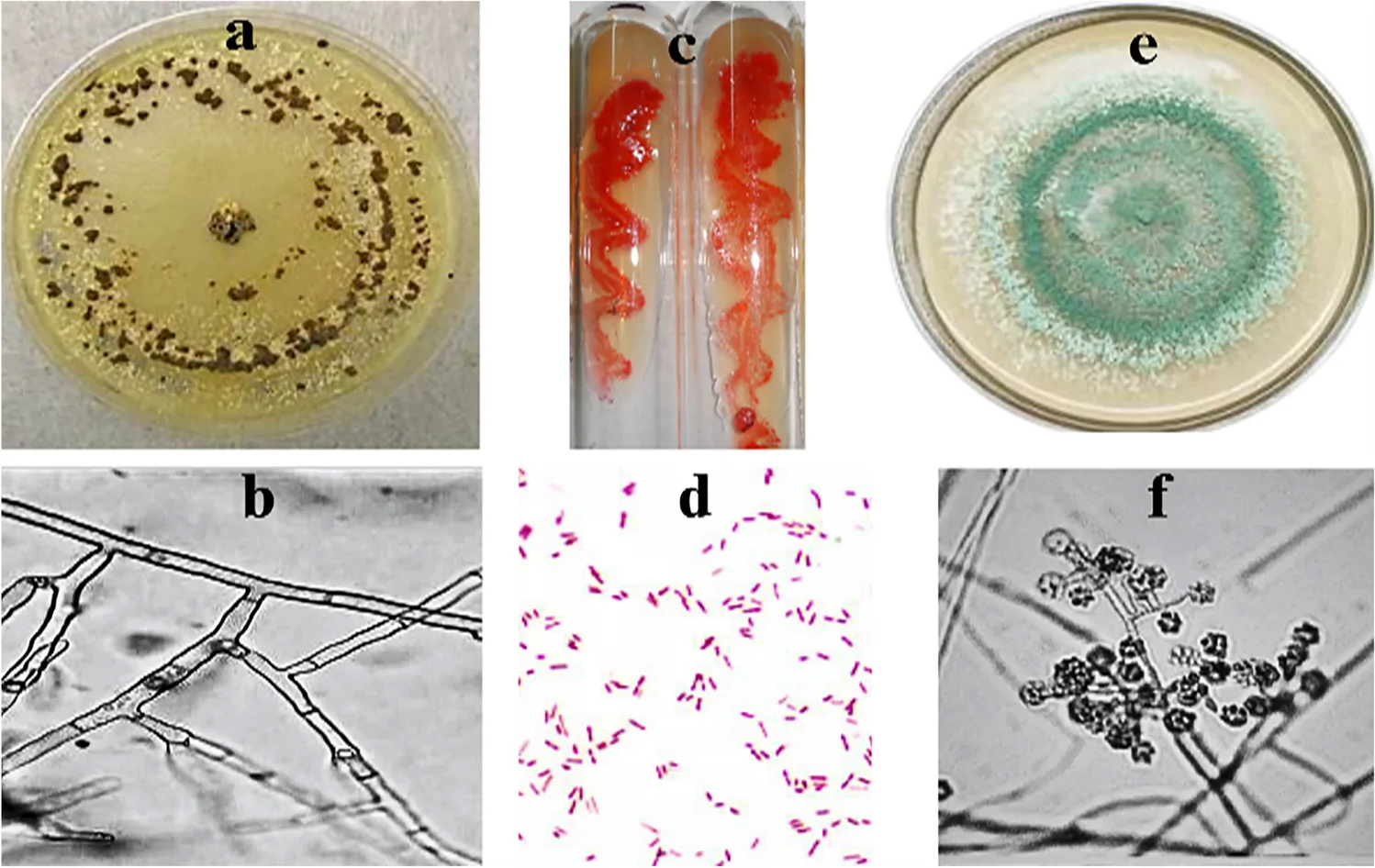
Colonies of the isolated bacterial and fungal isolates. **a**: R4 fungal isolate and **b**: microscopic examination of R4 isolates indicating the hyphae branch at right angles or acute angles. **c**: S1 bacterial isolate and **d**: microscopic examination of S1 isolate indicating Gram-negative short rods. **e**: T15 fungal isolate indicating greenish colonies with branching or simple conidiophores in brush-like cluster. **f**: Microscopic examination of T15 isolate indicating green or yellow-green in color conidiospores and are borne in clusters at the tips of the conidiophores

The bacterial isolate, S1, had a round colony with smooth surface end edges and red color. Microscopic examinations confirmed its shape and motility as it had a short rod shape and was non-motile. Fungal isolate R4 had black conidial spores while T15 isolate exhibited greenish colonies with branching or simple conidiophores in brush-like clusters as shown in (Fig. 5).

#### Molecular Identification

**Fig. 6:**
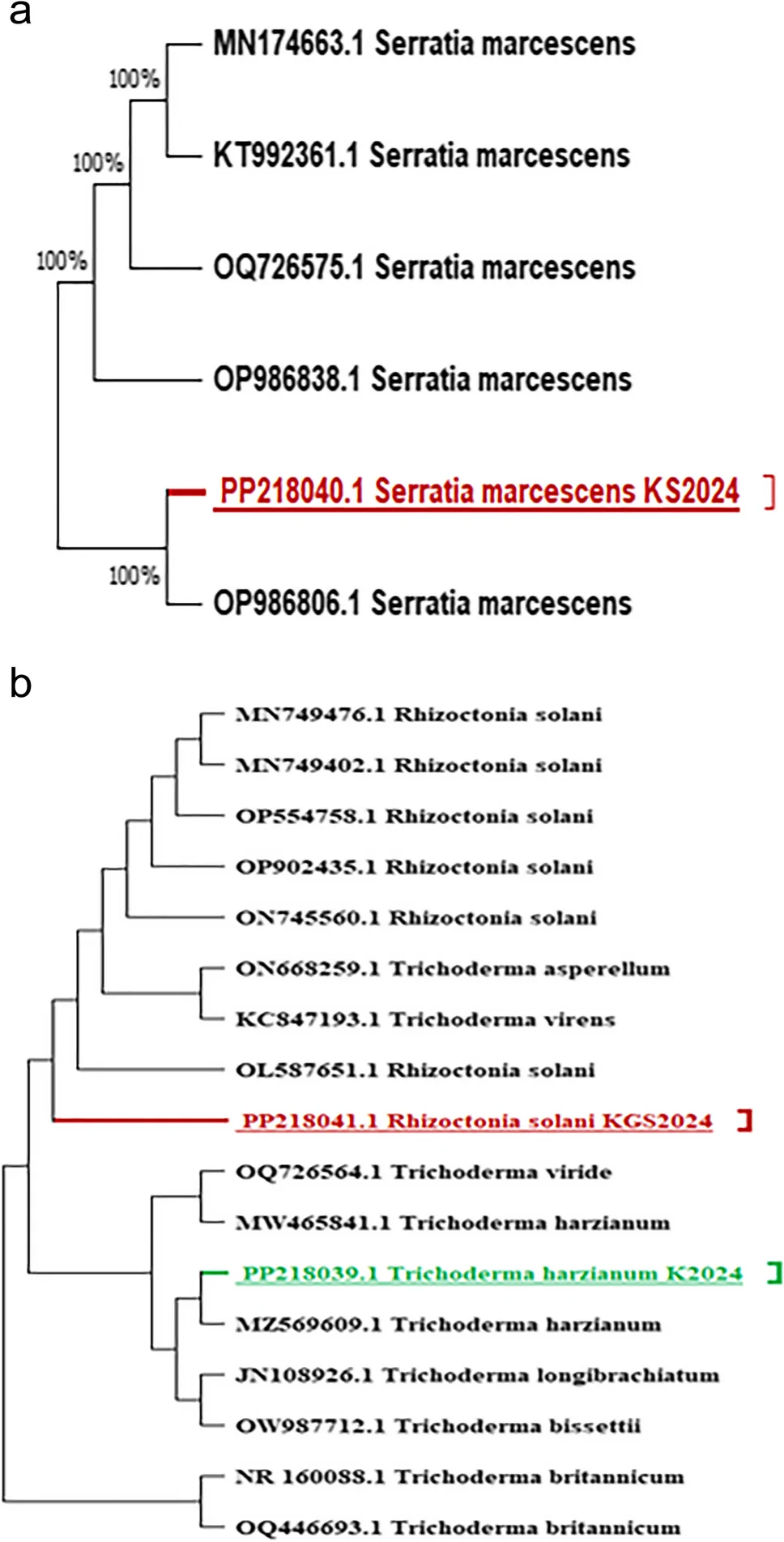
**a**: Neighbor-joining tree based on 16S rRNA sequences obtained from BLAST search indicating the position of *Serratia marcescens* isolate and related strain. **b**: Neighbor-joining tree based on 18S rRNA sequences obtained from BLAST search indicating the position of *Rhizoctonia solani* and *Trichoderma harzianum* isolates and related strain

16S rRNA and 18S rRNA gene sequence analysis were used to identify the predominant unknown fungal isolate that showed deteriorative activity for common bean cv. Giza 6 plants and the obtained biocontrol agents. The neighbor-joining algorithm was used to assemble the phylogenetic tree shown in (Fig. 6a and b). In the bacterial phylogenetic tree, *Serratia marcescens* 16S rRNA sequence types existed. The results illustrated in Fig. 6a showed that the 16S rRNA gene sequence was consistent with that of (NCBI, Bethesda, MD, USA); thus, it was identified as *S. marcescens* KG2024 (accession No. PP218040). Furthermore, the affinity between the isolates and their nearest phylogenetic neighbors is shown in Fig. 6b, which includes a comparison of the sequence information for several *Serratia* isolates. The findings revealed an almost 98% sequence similarity between the 5 *Serratia* spp. and *S. marcescens*. In the fungal phylogenetic tree, isolates belonging to *Trichoderma harzianum KGS2024* (accession No. PP218039) and *Rhizoctonia solani* KS2024 (accession No. PP218041) were identified.

### Bioagents Trials Under Greenhouse Conditions

Greenhouse trials were conducted to evaluate combinations of vermicompost tea (VCT), effective microorganisms (EM1), *Trichoderma harzianum*, and *S. marcescens* against *R. solani* infection in common bean cv. Giza 6. Disease incidence in terms of pre-emergence, post-emergence damping-off, and plant survival along with treatment efficacy percentage are presented (Table 1). All the antagonistic treatments significantly reduced both pre- and post-emergence mortality over pathogen control. Combinations mostly showed higher disease suppression than individual applications. At 5% VCT, EM1 + VCT and *Trichoderma harzianum* + VCT showed maximum efficacy of 94.54% and 94.51% respectively in improving plant stand. With 10% VCT, EM1 + VCT gave 94.51% efficacy followed by 83% for *Trichoderma harzianum* + VCT. *S. marcescens* alone had the lowest 43.89% efficacy.

**Table 1:** Effect of different biocontrol treatments on damping-off parameters and efficacy percentage against *R. solani* root rot in common bean under greenhouse conditions

Treatments		Pre-emergence damping-off %	Post-emergence damping-off %	Survival %	Efficiency
VCT 5% (V/W)	VCT	10.03D ± 0.26	11.10G ± 0.17	78.87	76.93
	+ *Trichoderma harzianum*	5.03E ± 0.49	0.00I ± 0.0	94.97	94.51
	+ *S. marcescens*	5.03E ± 0.20	15.57E ± 0.13	79.40	77.51
	+ EM1	5.00E ± 0.28	0.00I ± 0.0	95.00	94.54
VCT 10% (V/W)	VCT	15.03C ± 0.31	17.63D ± 0.20	67.34	64.34
	+ *Trichoderma harzianum*	10.07D ± 0.29	5.50H ± 0.20	84.43	83.00
	+ *S. marcescens*	10.03D ± 0.20	5.63H ± 0.20	84.34	82.90
	+ EM1	5.03E ± 0.26	0.00I ± 0.0	94.97	94.51
*Trichoderma harzianum*		15.03C ± 0.26	29.40C ± 0.17	55.57	51.50
*S. marcescens*		20.07B ± 0.29	31.33B ± 0.31	48.60	43.89
EM1		15.03C ± 0.20	11.77F ± 0.14	73.20	70.74
Fungicide (Rizolex-T 50%)		10.03D ± 0.37	5.63H ± 0.31	84.34	82.90
Control (autoclaved soil)		0.00F ± 0.0	0.00I ± 0.0	100.00	100.00
Control (infested soil)		24.97A ± 0.26	66.63A ± 0.20	8.40	0.00

Values are the averages of 3 replicates ± SD. Different letters indicate significant differences, according to Tukey’s Studentized Range (HSD) Test (*P* < 0.05)

### Bioagents Trials Under Field Conditions

#### Disease Severity

**Fig. 7:**
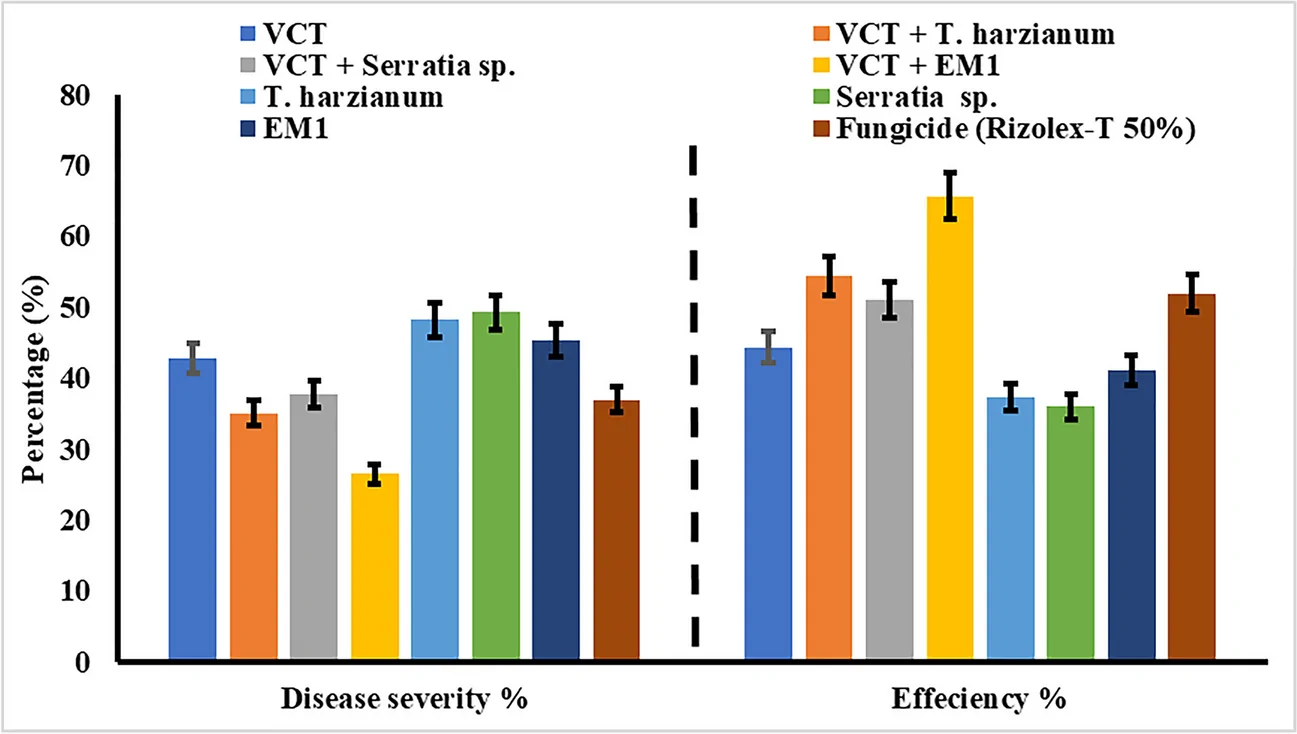
Effect of single and different combinations of treatments on root rot disease severity on common bean cv. Giza 6 under field conditions

Field trials were conducted to evaluate combinations of vermicompost tea (VCT), effective microorganisms (EM1), *Trichoderma harzianum*, and *S. marcescens* against *R. solani* infection in common bean cv. Giza 6. The comparison among different treatments reveals variations in their efficacy in controlling disease severity and promoting plant health against damping-off in common bean plants as illustrated in (Fig. 7). Treatments combining vermicompost tea (VCT) with biological agents like *Trichoderma harzianum*, *S. marcescens*, and EM1 demonstrate superior performance compared to individual treatments or chemical fungicide applications. Particularly, the combination of VCT with EM1 stands out, exhibiting the lowest disease severity percentage at 26.47% (± 0.38) and the highest efficiency percentage at 65.60%. Conversely, treatments solely relying on *Trichoderma harzianum* or *S. marcescens* show higher disease severity percentages and lower efficiency percentages, indicating their limited effectiveness in mitigating damping-off. Chemical fungicide application, while moderately effective with a disease severity percentage of 36.97% (± 0.84) and an efficiency percentage of 51.95%, falls short compared to the combined VCT and biological agent treatments. These findings underscore the potential of integrated approaches combining organic amendments like VCT with compatible biological agents in effectively managing damping-off and enhancing plant resilience.

### Growth Parameters

#### Pod Weight

**Fig. 8:**
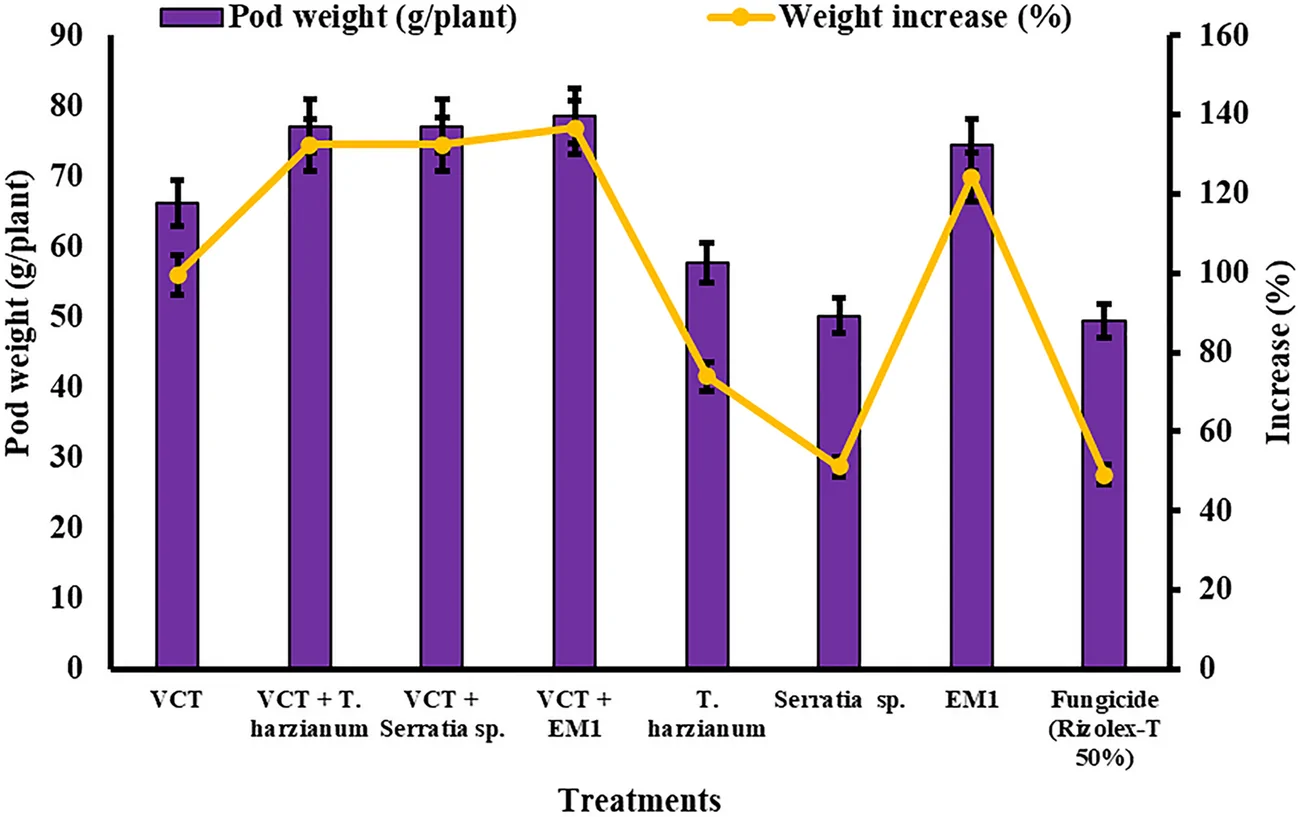
Effect of single and different combinations of treatments on common bean cv. Giza 6 pod weight/plant under field conditions

In Fig. 8, all treatments demonstrated a noticeable increase in pod weight per plant compared to untreated plants, highlighting their efficacy in enhancing common bean cv. Giza 6 yield under field conditions. The most substantial improvement in pod weight per plant was observed with the VCT + EM1 treatment, exhibiting a remarkable increase of 136.68%, followed closely by the VCT + *S. marcescens* combination, which achieved a 132.49% increase. Notably, all treatments surpassed the pod weight per plant enhancement achieved by the Fungicide (Rizolex-T 50%) treatment, which yielded a 49.10% increase compared to untreated plants. Specifically, VCT + *Trichoderma harzianum* and VCT + *S. marcescens* combinations demonstrated comparable increases in pod weight per plant at 132.34% and 132.49%, respectively. The treatments involving individual biological agents, such as *Trichoderma harzianum*, *S. marcescens*, and EM1, also contributed to notable increases in pod weight per plant, ranging from 51.18 to 124.35%. Overall, the findings underscore the significant potential of these treatments in augmenting common bean yield and suggest the superiority of combined treatments, particularly those incorporating VCT and biological agents, in maximizing pod weight per plant under field conditions. The significance of treatment effects was confirmed at *P* ≤ 0.05.

#### Shoot Length

**Fig. 9:**
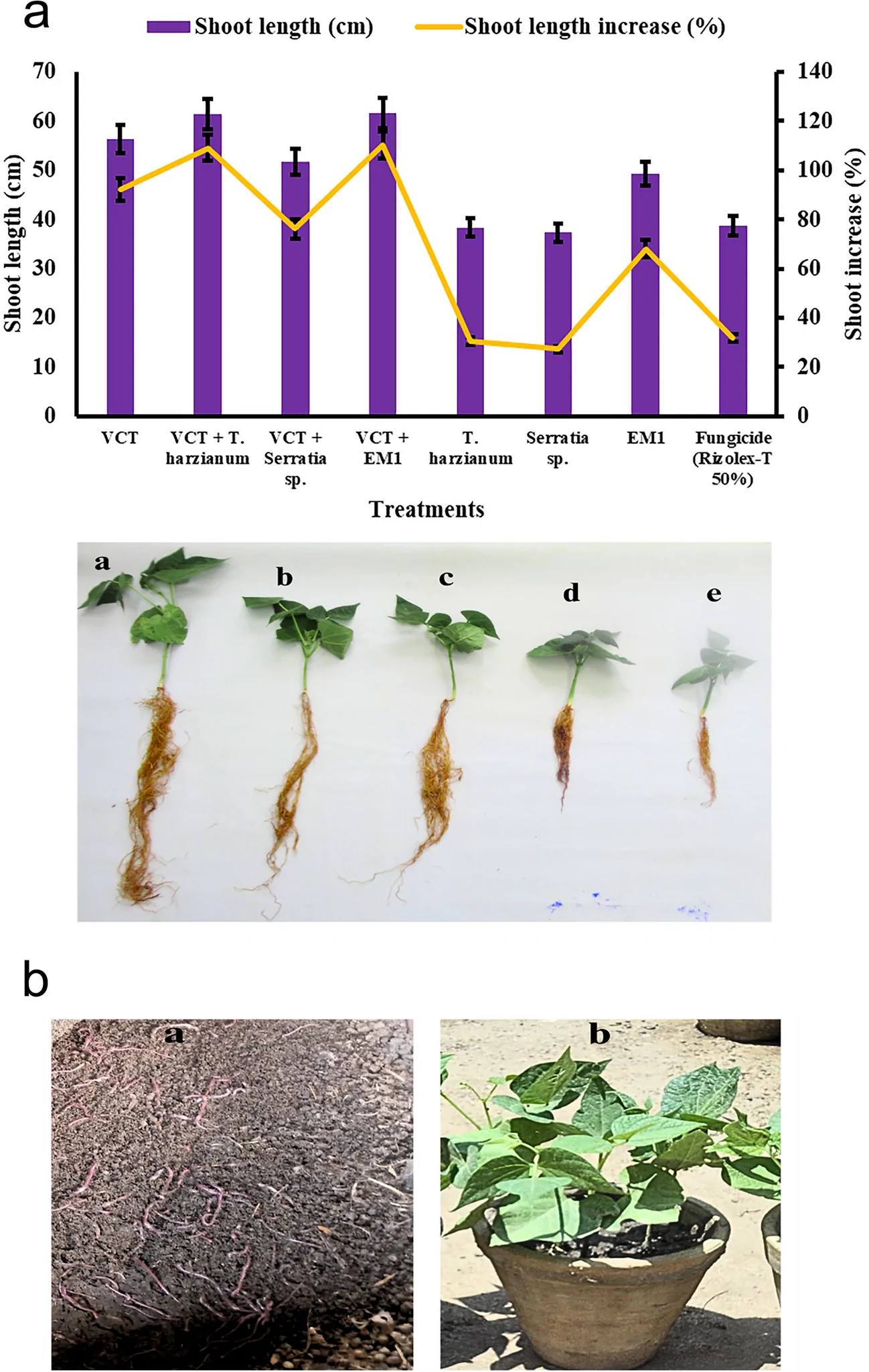
**a**: Effect of single and different combinations of treatments on shoot length and root length of common bean cv. Giza 6 plants under field conditions. (a) VCT + *T. harzianum*, (b) VCT + EM, (c) VCT + *Serratia marcescens*, (d) EM1. **b**: Effect of vermicompost on common bean plant. The first image (a) appears to show vermicompost, which is a nutrient-rich soil amendment produced by the breakdown of organic matter by worms or other decomposer organisms before soaking treatment. The second image (b) depicts a healthy, lush potted plant, likely benefiting from compost amendment, vermicompost

As demonstrated in Fig. 9a and b, all treatments resulted in increased shoot length and root length compared to untreated plants, indicating their positive impact on the growth of common bean cv. Giza 6 plants under field conditions. The VCT + EM1 treatment notably excelled in enhancing shoot length, yielding a significant increase of 110.26%, followed closely by the VCT + *Trichoderma harzianum* combination, which achieved a growth percentage of 109.10%. Conversely,* S. marcescens* exhibited the least effect on shoot length with a growth percentage of 27.28%. Similarly, the trends in root length mirrored those of shoot length, with the VCT + EM1 treatment significantly boosting root length by 136.59%, followed by VCT + *Trichoderma harzianum* with an increase of 82.75%. *S. marcescens* again showed the least impact on root length, with a growth percentage of 17.31%. These findings underscore the efficacy of combined treatments, particularly those incorporating VCT and EM1, in promoting robust shoot and root growth in common bean plants. The significance of treatment effects was confirmed at *P* ≤ 0.05.

#### Shoot, Root, and Dry Weight

**Fig. 10:**
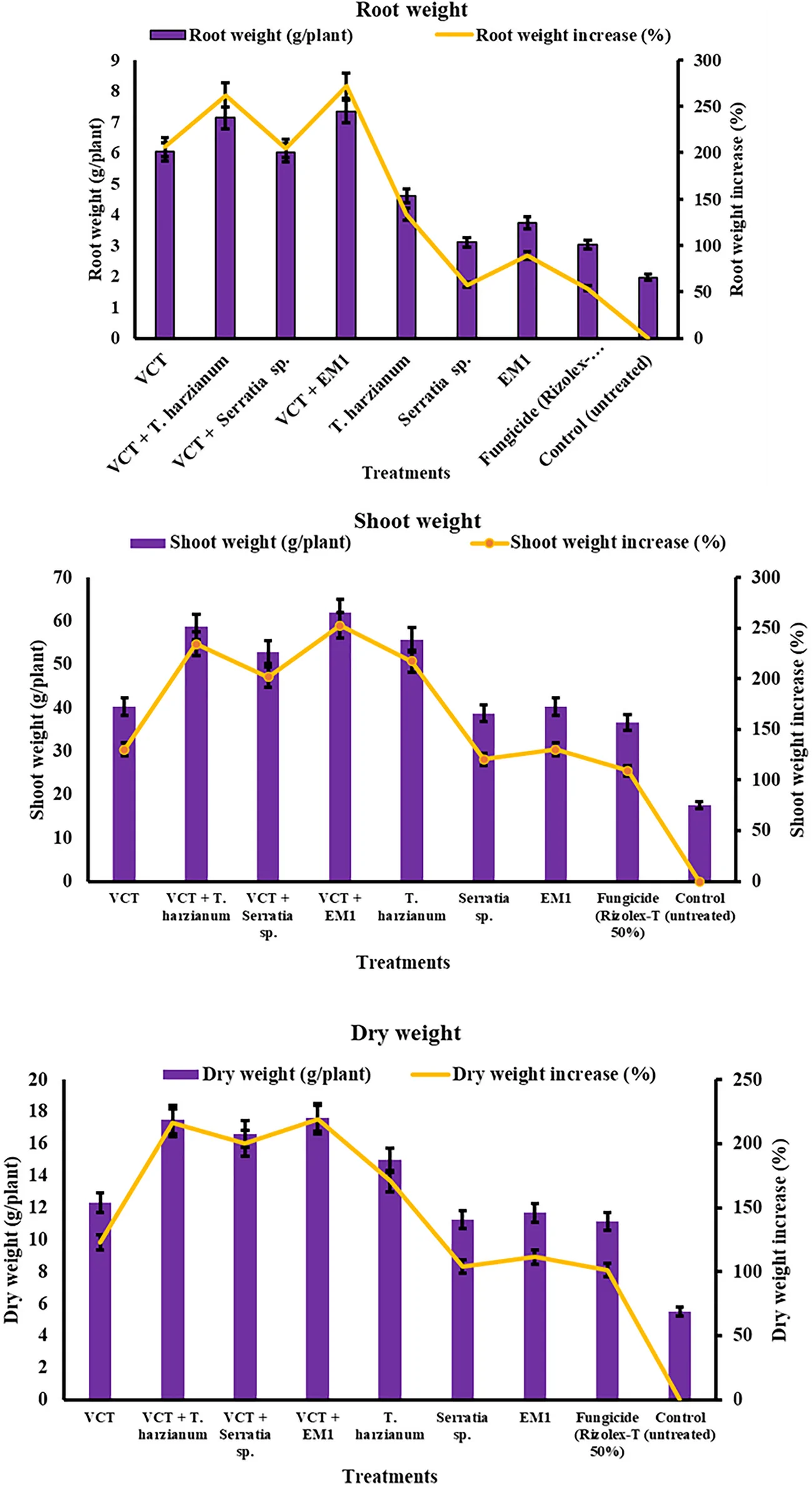
Effect of single and different combinations of treatments on root weight, shoot weight, and dry weight of common bean cv. Giza 6 plants under field conditions

As demonstrated in Fig. 10, all treatments led to increased shoot weight, root weight, and dry weight compared to untreated plants, reflecting their positive effects on the growth and development of common bean plants. The VCT + EM1 treatment notably excelled in enhancing plant parameters, significantly increasing shoot weight by 252.49%, root weight by 272.08%, and dry weight by 218.81% compared to untreated plants. Conversely, *S. marcescens* exhibited the least impact, with increases of 120.59% in shoot weight, 57.36% in root weight, and 103.80% in dry weight compared to non-treated plants. The combined treatment of VCT + *Trichoderma harzianum* also demonstrated substantial enhancements, with increases of 234.68% in shoot weight, 261.93% in root weight, and 216.46% in dry weight. Treatments involving individual agents, such as *Trichoderma harzianum* and *S. marcescens*, showed moderate improvements across parameters, while the control group exhibited the lowest values. These findings underscore the effectiveness of treatments, particularly those combining VCT and EM1, in significantly promoting shoot, root, and dry weights, thereby enhancing overall plant growth and productivity.

### Peroxidase and Polyphenol Oxidase Enzyme Activities

**Fig. 11:**
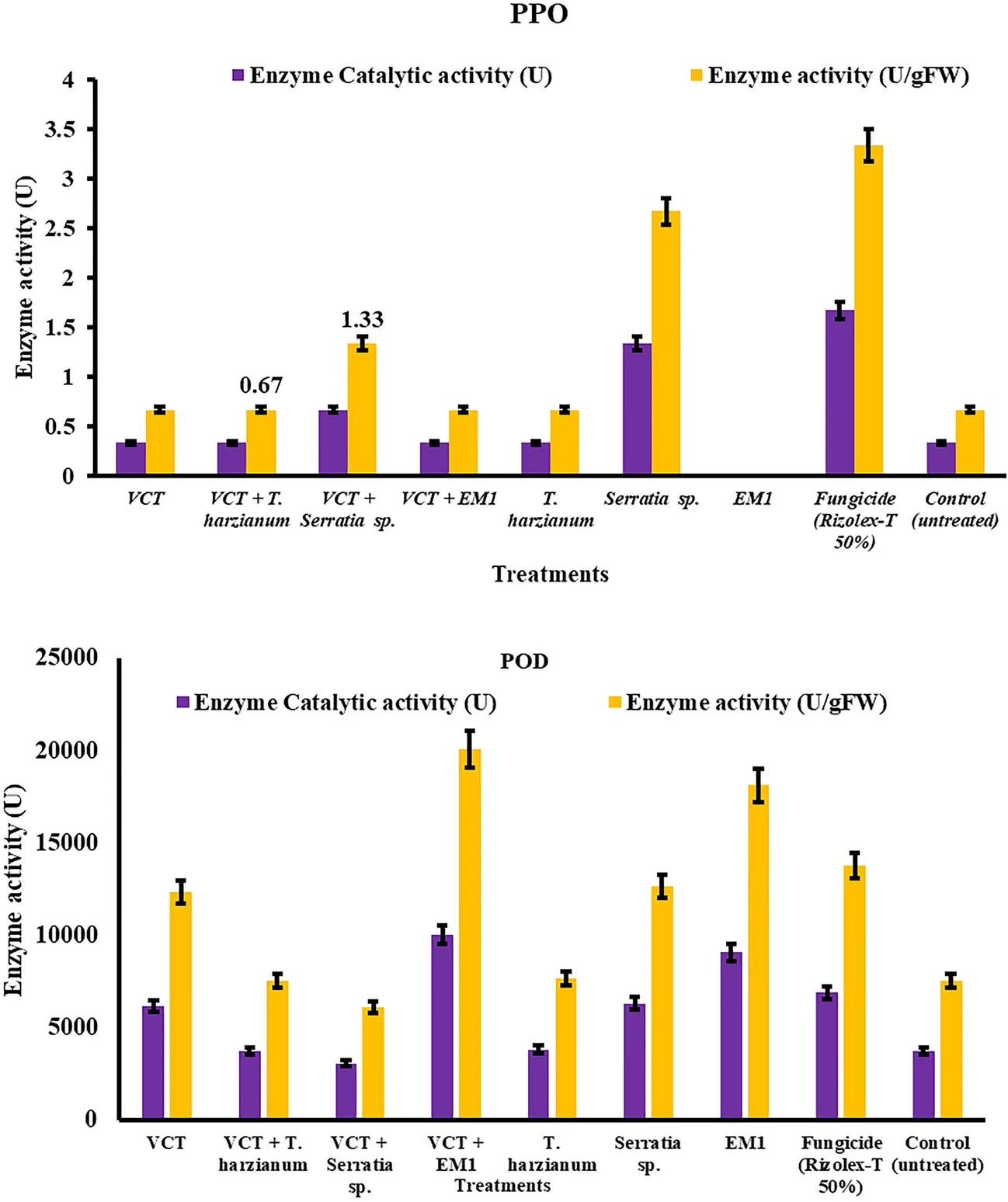
Peroxidase (POD) and polyphenol oxidase activities, expressed as a rate of hydrogen peroxide conversion per fresh mass (FM) in common bean cv. Giza 6 leaves as influenced by different treatments

The effect of vermicompost tea, EM1, and bioagents on the activity of peroxidase and polyphenol oxidase enzyme of leaves from common bean cv. Giza 6 plants infected with *R. solani* root rot was studied, and the data was obtained. Results presented in Fig. 11 show that most treatments increased the activity of peroxidase enzyme (U/gfw) and some treatments increased the activity of polyphenol oxidase enzyme (U/gfw) in common bean-infected leaves. The higher content peroxidase was recorded by EM1 + VCT, EM1, *S. marcescent*, and *Trichoderma harzianum* respectively in common bean-infected leaves. The lowest content was recorded in *S. marcescens* + VCT in common bean-infected leaves.

### Gas Chromatography (GC–MS) Analysis

The GC–MS analysis identified several abundant compounds (Table 2) in the *Trichoderma harzianum* culture, including cyclic pregnane (7.57%), hexadecanoic acid methyl ester (53.27%), 9-octadecenoic acid methyl ester (23.75%), 6-octadecenoic acid methyl ester (3.11%), and octadecanoic acid methyl ester (4.64%). Minor compounds were also found such as tetradecanoic acid methyl ester (4.64%), cyclopentaneundecanoic acid methyl ester (4.64%), 1,3-benzenedicarboxylic acid (5.79%), and some octadecadiynoic acid derivatives (1.87%). For *S. marcescens*, the main compounds found were 9,12-octadecadienoic acid derivatives like the 2-hydroxy-1-(hydroxymethyl)ethyl ester (0.63%) and linoleic acid (4.20%), along with two hexadecadienoic acid related compounds (0.59%). Finally, the VCT sample predominantly contained the four most important compounds which are hexadecanoic acid, methyl ester with (37.51%); 9-octadecenoic acid (Z)-, methyl ester (44.00%); octadecanoic acid, methyl ester (4.19%); and 9,12-octadecadienoic acid (Z, Z)-, 2,3-bis[(trimethylsilyl)oxy]propyl ester (5.62%).

**Table 2:** The most abundant compounds detected in VCT extract and metabolites of *S. marcescent* and *Trichoderma harzianum* using GC/MS

Efficient compounds	No	RT (min)	Area (%)	Library/ID
*Trichoderma harzianum*	1	4.05	7.57	PREGNANE-3,11,20,21-TETROL, CYCLIC 20,21-(BUTYL BORONATE), (3à,5á,11á,20R)-
	2	4.05	7.57	DIMETHYLDIPHENYLTETHYLIDYLPYRROLIDINE
	3	4.05	7.57	1-{2-[(2-ISOPROPYL-5-METHYLCYCLOHEXYL)OXY]-2-OXOETHYL}-2-(4-METHOXYPHENYL)-3-METHYL-3H-BENZIMIDAZOL-1-IUM
	4	4.05	7.57	1-Di(tert-butyl)silyloxy-2-phenylethane
	5	23.23	53.27	Hexadecanoic acid, methyl ester
	6	26.41	23.75	9-Octadecenoic acid (Z)-, methyl ester
	7	26.52	3.11	6-Octadecenoic acid, methyl ester, (Z)-
	8	26.88	4.64	OCTADECANOIC ACID, METHYL ESTER
	9	26.88	4.64	Tetradecanoic acid, 12-methyl-, methyl ester
	10	26.88	4.64	Cyclopentaneundecanoic acid, methyl ester
	11	36.52	5.79	1,3-Benzenedicarboxylic acid, bis(2-ethylhexyl) ester
	12	39.58	1.87	2,5-Octadecadiynoic acid, methyl ester
	13	39.58	1.87	Cholesta-8,24-dien-3-ol, 4-methyl-, (3á,4à)-
*S. marcescens*	1	28.58	0.63	9,12-Octadecadienoic acid (Z,Z)-, 2-hydroxy-1-(hydroxymethyl)ethyl ester
	2	29.51	4.20	9,12-Octadecadienoic acid (Z,Z)-
	3	40.53	0.59	Hexadecadienoic acid, methyl ester
	4	40.53	0.59	Linoleic acid ethyl ester
VCT	1	25.60	37.51	Hexadecanoic acid, methyl ester
	2	28.78	44.00	9-Octadecenoic acid (Z)-, methyl ester
	3	29.34	4.19	Octadecanoic acid, methyl ester
	4	42.81	5.62	9,12-Octadecadienoic acid (Z, Z)-, 2,3-bis[(trimethylsilyl)oxy]propyl ester

## Discussion

The isolation of *Rhizoctonia* sp., *Fusarium* sp., *Macrophomina* sp., and *Pythium* sp. from infected common bean plants in this study aligns with previous reports identifying these fungi as major causal agents of root rot diseases in common beans [18]. *Rhizoctonia solani* was the predominant isolate at 50% frequency, conferring high virulence in the pathogenicity assays, consistent with its recognition as a destructive pathogen inflicting substantial yield losses in common beans [22].

The antagonistic activity demonstrated in vitro by *Serratia* sp. S1 and *Trichoderma* sp. T15 isolates against *R. solani* is supported by earlier works documenting the biocontrol efficacy of these genera against phytopathogens through mechanisms like antibiosis, enzyme secretion, and induced resistance [32, 33]. The 54.2% and 65.2% growth inhibition of *R. solani* achieved respectively aligns with inhibitions of 40–60% reported for *Serratia* spp. and 60–100% described for *Trichoderma* spp. The superior antagonism of 10% VCT, yielding inhibition zone diameters up to 3.5 mm, can be attributed to its enrichment in diverse microbial communities and metabolites, as noted previously [34].

The identification of the bacterial isolate S1 as *S. marcescens* via 16S rRNA gene sequencing corroborates earlier works recognizing *S. marcescens* as an eminent biocontrol agent against fungal phytopathogens like *R. solani* and *F. oxysporum*, mediated through chitinase production, siderophore secretion, and induced systemic resistance pathways [35]. *Trichoderma harzianum* is also a well-established biocontrol fungus applied against soil-borne diseases, as confirmed by 18S rRNA sequencing of isolate T15 [36].

Under greenhouse conditions, combinations of VCT and the antagonists *S. marcescens*, *Trichoderma harzianum*, or EM1 conferred 94.51–94.54% efficacy in improving plant stand by suppressing both pre- and post-emergence damping off incited by *R. solani* up to 95%, outperforming their applications. These findings concur with previous results demonstrating synergistic effects between vermicompost/compost teas and biocontrol agents like *Trichoderma* sp. and plant growth-promoting rhizobacteria, enhancing biocontrol efficacy against fungal pathogens by up to 80% in beans and other crops [13, 37, 38].

The integrated treatments also proved more effective under field conditions, with VCT + EM1 and VCT + *Trichoderma harzianum* lowering disease severity by 65.6% and 64.34% respectively, coupled with at least 100% increases in growth parameters like pod weight, shoot length, root length, and dry weight. The 136.68% augmentation of pod weight per plant achieved with VCT + EM1 translates to pronounced yield gains, consistent with earlier reports of vermicompost and EM-fermented organic amendments elevating common bean and vegetable yields by 75–150% over chemical fertilizers [25]. The remarkable efficacy of VCT-based combinations can be ascribed to the diverse microbial communities and plant growth-promoting metabolites conferred by vermicomposts [39].

The enzyme assay results revealed general increases in peroxidase activity in bean leaves in response to the treatments, considered an important marker of systemic acquired resistance against pathogens [29, 40]. The highest peroxidase induction by EM1 and EM1 + VCT aligns with findings describing EM formulations as strong elicitors of defense enzyme activity in various crops [41].

The GC–MS profiling revealed an array of bioactive secondary metabolites and derivatives in the tested microbial and vermicompost tea extracts. The predominance of cyclic pregnane (7.57%), various fatty acid methyl esters (FAMEs) like hexadecanoic and octadecanoic acid forms (53.27% and 4.64% respectively), and minor quantities of aromatic organic acids (5.79%) among key compounds detected in *Trichoderma harzianum* align well with earlier reports describing such molecules as major components of *Trichoderma* sp. exometabolome with confirmed antifungal, antibacterial, and plant growth-promoting functions [30, 42, 43]. Similarly, linoleic acid (4.20%) and its hydroxy derivatives found in *Serratia marcescens* extract have established roles in biocontrol activity against phytopathogen [42, 44]. The high levels of beneficial free fatty acids especially oleic, palmitic, linoleic, and stearic forms noted in VCT (55.82 to 3.08%) substantiate previous studies demonstrating enrichment of such plant growth-supporting nutrients in vermicompost tea metabolites [20].

The antimicrobial compounds produced by the bacterial strains *Enterobacter cloacae* PM23 and *Bacillus mycoides* PM35 play a crucial role in their multi-stress resistance potential [45]. The antibiotic-resistant Iturin C (ItuC) and bio-surfactant-producing genes (sfp and srfAA) amplified in *E. cloacae* PM23 contribute to its ability to resist biotic stresses. Similarly, *B. mycoides* PM35 possesses genes conferring abiotic stress tolerance (CzcD, sfp, and srfAA genes), which enhance its resilience against environmental stresses [46]. These antimicrobial compounds and biosurfactants can disrupt the cell membranes of competing microorganisms and pathogens, inhibiting their growth and providing a biocontrol mechanism for plant protection. Additionally, the production of antibiotics by these bacterial strains can directly inhibit or kill phytopathogenic bacteria, fungi, and other harmful microbes, thereby safeguarding plant health [47].

Beneficial bacteria, particularly endophytic bacterial communities, employ various mechanisms to control plant pathogens and promote plant growth [48]. These endophytic bacteria play a crucial role in plant growth promotion (PGP) by regulating indirect mechanisms targeting pests and pathogens. They can produce hydrolytic enzymes, antibiotics, and other biocontrol compounds that inhibit or kill phytopathogenic microorganisms. Additionally, these bacteria can compete with pathogens for nutrients and space, restricting their growth and proliferation within plant tissues [49]. Some endophytic bacteria can also induce systemic resistance in plants, enhancing their defense mechanisms against pathogens [50]. Furthermore, the document emphasizes the importance of detecting and diagnosing plant bacterial diseases through various techniques, including conventional serological, observation-based, and molecular methods, as well as emerging biosensor-based techniques, point-of-care (POC) systems, robotics, and cell phone-based systems. The early and accurate detection of plant pathogens is crucial for implementing effective management strategies and minimizing crop losses as well as the combined application of Staphylococcus and zinc oxide nanoparticles boosted wheat growth, physiology, defense system, and reduced chromium accumulation [51].

Various eco-friendly strategies were highlighted, including using beneficial microorganism plant growth-promoting rhizobacteria (PGPR), biosensors, and floating treatment wetlands for phytoremediation and disease diagnosis. In line with these approaches, phosphate solubilizing bacteria (PSB) and organic amendments improved wheat growth, phosphorus availability, and soil acidification in calcareous soils [52]. Floating treatment wetlands using plants and bacteria were reviewed for remediating petroleum hydrocarbon-contaminated water cost-effectively and eco-friendly [53]. The sustainable management of plant diseases and promotion of plant growth are crucial for achieving agricultural sustainability and food security.

Overall, the results validate the integration of VCT with biocontrol microbes like *S. marcescens*, *Trichoderma harzianum*, and EM consortia as a sustainable alternative to chemical fungicides for effectively managing *R. solani*-induced root rot in common beans, with potential utility against other soil-borne diseases in diverse cropping systems. The pronounced yield increases demonstrate the multifunctional benefits of augmenting soil quality through vermicompost-based biologics for healthier plant growth.

## Conclusion

The results of this study demonstrate the potential of an integrated approach using vermicompost tea (VCT) and beneficial microbes like *Serratia marcescens*, *Trichoderma harzianum*, and effective microorganisms (EM1) for effective management of Rhizoctonia root rot in common beans. The combinations of 10% VCT with the antagonistic isolates displayed strong inhibitory activity against the virulent pathogen *R. solani* in vitro. Moreover, applications of 5–10% VCT together with *S. marcescens*, *Trichoderma harzianum*, or EM under greenhouse conditions afforded up to 95% suppression of damping-off disease incidence in bean seedlings caused by *R. solani*. The integrated treatments translated to significant disease control and growth enhancements under field conditions as well. Particularly, the VCT + EM treatment lowered disease severity by 65.6% and increased pod weight per plant by 136.68% over unprotected plants, indicating substantial yield gains. The treatments also elicited defense enzyme activity in bean leaves while promoting robust improvements in shoot length, root length, and dry biomass. The remarkable efficacy demonstrates synergistic interactions between the rich microbial consortia and bioactive compounds provided by VCT and the selected antagonists, with potential involvement of mechanisms like induced systemic resistance, enzymatic parasitism, and nutrient competition. Overall, the results validate VCT-mediated delivery of disease-suppressive microorganisms as a sustainable platform for integrated management of debilitating root rot diseases in beans. By regenerating soil health and resilience, this climate-smart approach can foster productivity and incomes for smallholder farmers. Further validation can accelerate adoption for strengthening food and nutrition security across developing countries.

## Data Availability

The raw data and analyzed data used during the current study are available from the corresponding author on reasonable request. All the isolated microorganisms were identified using 16 s rRNA gene analysis and deposited in the GenBank as follows: 1 *Serratia marcescens* https://www.ncbi.nlm.nih.gov/nuccore/PP218040 2 *Trichoderma harzianum* https://www.ncbi.nlm.nih.gov/nuccore/PP218039 3 *Rhizoctonia solani* https://www.ncbi.nlm.nih.gov/nuccore/PP218041
